# Complete Genome Sequence of *Mycobacterium tuberculosis* Strain MtURU-001, Isolated from a Rapidly Progressing Outbreak in Uruguay

**DOI:** 10.1128/genomeA.01220-13

**Published:** 2014-01-23

**Authors:** Gonzalo Greif, Gregorio Iraola, Luisa Berná, Cecilia Coitinho, Carlos M. Rivas, Hugo Naya, Carlos Robello

**Affiliations:** a Unidad de Biología Molecular, Institut Pasteur de Montevideo, Montevideo, Uruguay; b Unidad de Bioinformática, Institut Pasteur de Montevideo, Montevideo, Uruguay; c Sección Genética Evolutiva, Facultad de Ciencias, Universidad de la República, Montevideo, Uruguay; d Comisión Honoraria de Lucha Antituberculosa y Enfermedades Prevalentes, Montevideo, Uruguay; e Departamento de Producción Animal y Pasturas, Facultad de Agronomía, Universidad de la República, Paysandú, Uruguay; f Departamento de Bioquímica, Facultad de Medicina, Universidad de la República, Montevideo, Uruguay

## Abstract

Despite efficient control programs, large clonal outbreaks of tuberculosis (TB) may arise in low-risk populations. Recently, an unusual TB outbreak was reported in Uruguay, reaching an elevated disease attack rate (53 to 69%). Here, we report the genome sequence of the *Mycobacterium tuberculosis* strain associated with this rapidly progressing outbreak, named MtURU-001.

## GENOME ANNOUNCEMENT

Recently, we reported an unusual tuberculosis (TB) outbreak centered on a professional basketball team in Montevideo, Uruguay, a country with a low TB incidence (1). In August 2008, a young male member of the basketball team was diagnosed with TB, and a chest X-ray indicated a bilateral pulmonary form with cavities. TB was bacteriologically confirmed 20 days later and the patient was cured after first-line treatment. As described in Coitinho et al. (1), following this index case, six other team members who lived at the same place for a week and four other contacts were successively diagnosed with TB over the next 2.5 years. All patients (ranging between 17 and 23 years of age) were immunocompetent, athletic, and wealthy. No other comorbidity factors were detected.

Despite control programs, large clonal TB outbreaks can develop even in low-incidence countries, reflecting ongoing disease transmission (2, 3). The *Mycobacterium tuberculosis* strain showed an elevated disease attack rate (53 to 69%) that sharply contrasts with the lifetime risk of developing active TB, being estimated at <10% among infected individuals in the general population. We report here the draft genome sequence of the TB isolate from the index case.

Sequencing was performed at the Institut Pasteur de Montevideo on an Illumina Genome Analyzer IIx platform and generated 2,379,897 paired-end reads (2 × 100 cycles). The resulting library was first corrected using ALLPATHS-LG (4) and then assembled with Velvet (5) software, producing 195 contigs with an average coverage of 84-fold. The assembly quality was improved using the PAGIT toolkit (6), based on the genome sequence of *M. tuberculosis* H37Rv (GenBank accession no. NC_000962) as a reference strain. The final assembly quality was evaluated with the Assembly Likelihood Estimator (ALE) software (7), and the assembly was automatically annotated with RAST (8).

*M. tuberculosis* MtURU-001 has a circular chromosome of 4,378,296 bp, with an average G+C content of 65%, including 4,314 protein-encoding genes, 1 rRNA operon, and 45 tRNA genes. In comparison with *M. tuberculosis* H37Rv, 4,096 orthologous groups were defined with OrthoMCL (9) and 1,016 polymorphisms were identified using the Burrows-Wheeler Aligner (BWA) (10) and GATK (11). A subset of 849 polymorphisms (802 single-nucleotide polymorphisms [SNPs] and 47 indels) were inside coding sequences, and 480 affect protein sequences, especially 24 that introduced stop codons disrupting several hypothetical proteins, one transcriptional regulator, 2 genes for the haloacid dehalogenase (HAD) superfamily, and 3 involved in lipid metabolism. Further comparative genomics across this genome may provide genotype-phenotype associations that might explain the rapid progression of this unusual outbreak.

### Nucleotide sequence accession numbers.

This whole-genome shotgun project has been deposited at DDBJ/EMBL/GenBank under the accession no. AZHK00000000. The version described in this paper is version AZHK01000000.
